# Miniaturization and 3D Printing of Bioreactors: A Technological Mini Review

**DOI:** 10.3390/mi11090853

**Published:** 2020-09-14

**Authors:** Spyridon Achinas, Jorn-Ids Heins, Janneke Krooneman, Gerrit Jan Willem Euverink

**Affiliations:** Faculty of Science and Engineering, University of Groningen, 9747 AG Groningen, The Netherlands; j.i.heins@student.rug.nl (J.-I.H.); j.krooneman@rug.nl (J.K.); g.j.w.euverink@rug.nl (G.J.W.E.)

**Keywords:** miniaturization, 3D printing, bioreactors, materials, process intensification

## Abstract

Many articles have been published on scale-down concepts as well as additive manufacturing techniques. However, information is scarce when miniaturization and 3D printing are applied in the fabrication of bioreactor systems. Therefore, garnering information for the interfaces between miniaturization and 3D printing becomes important and essential. The first goal is to examine the miniaturization aspects concerning bioreactor screening systems. The second goal is to review successful modalities of 3D printing and its applications in bioreactor manufacturing. This paper intends to provide information on anaerobic digestion process intensification by fusion of miniaturization technique and 3D printing technology. In particular, it gives a perspective on the challenges of 3D printing and the options of miniature bioreactor systems for process high-throughput screening.

## 1. Introduction

The pressure to reduce bioreactor costs and accelerate the bioprocess development in the biotech industry is ongoing and increasing [1]. The identification of optimal parameters for new biotechnological processes is a costly and time-consuming part of the development process, due to the multiple settings [2]. Since optimizing process conditions at a manufacturing scale are not practical and efficient, the development of miniaturized models that represent the performance of the industrial process is essential to achieve reliable process characterization [3]. Thus, parametric optimization through process screening is urgently needed for bioprocess development [2]. Miniature bioreactors are useful as such a process screening method, where multiple formulations or conditions are screened to identify the optimal set of values [4]. Notably, the integration of engineering and biological principles is essential for the scaling-down of bioreactors [5].

Similar to reactor performance in chemical research, in biochemical research, the term bioreactor performance is often used. However, a rapid scan of the literature elucidated that bioreactor performance is a well-known known concept, but it is never thoroughly explained [6,7]. In practice, the performance of a biochemical conversion process, i.e., the bioreactor performance, is mainly determined by the benefit/cost ratio [8]. For optimization purposes, the criteria for a high volume/ low value-added product are usually different from criteria used for the low volume/high value-added product. Thus, a bioreactor’s performance can be either evaluated by the yield of the desired product on the substrate (g product per g substrate), the productivity (g product per L reactor volume per hour) or the final titer (g product per L reactor volume) [9].

However, process performance usually connects to a particular market price of the product. On the other hand, there are also costs involved in the microbial conversion processes. The reduction of costs is often the main objective of biochemical engineering [8]. The bioreactor vessel is the core part of the bioreactor system. Within the bioreactor vessel, the solution is mixed to maintain a homogeneous solution, which affects the optimal performance. Different mixing methods [10,11] are used to obtain the perfect mixing by looking at the fluid flow, mixing rate and mixing time as parameters. Furthermore, the cultivation process needs to be carefully controlled. Sensors for pH, temperature, dissolved oxygen concentration, and foam are necessary to maintain optimal conditions for bacterial growth and/or synthesis of products. Contrary to conventional chemical reactors, bioreactors must provide a higher degree of control over process upsets and prevent contaminations by other microorganisms. These bioreactors are used to grow bacteria in a complex fluidic environment. Before newly found bacteria can be used in biotechnological applications, the bacteria first need to be characterized to determine in which environmental circumstances the bacteria will perform optimally. It is also important to know how the bacteria will react to the different situations that are applied by the operators.

Bioreactors can also be used for the production of cells where batch production is facilitated. The problem is that these batch-wise operated bioreactors have a lower output per volume per hour unit than with continuously operated bioreactors. On the other hand, continuously operated bioreactors have more risk involved, because the impact of the failure is higher. However, if there is a way to reduce the effect of failure in these types of bioreactors, then this can replace the batch-wise operated bioreactor. Lowering the impact of failure can be achieved by spreading the risk. Such an approach implies a system of many continuous bioreactors working independently. Hence, if one or more bioreactor becomes contaminated, it can be shut down, and all other bioreactors remain in operation. The approach in scaling-up by numbers instead of scaling-up by size reduces the risks in fermentation processes but increases the amount of materials needed.

Biotechnological applications can differ significantly from each other. Therefore, a suitable process screening system varies per specific application. A flexible and modular system deals with the variation in applications. The 3D printing process helps to quickly manufacture the particular modules that are necessary for the screening. Additive Manufacturing describes the 3D printing process in a larger whole. The 3D printing is a crucial part of Additive Manufacturing as it enables the construction of the design layer-by-layer rather than through molding or subtractive techniques [12]. Moreover, this technique provides high customizability while producing small quantities at relatively low cost in a short period [13].

This paper combines technological perspectives of fusion of miniaturization and 3D printing for the development of multi-parallel bioreactor screening systems. This paper is a short and comprehensive perspective of 3D printing technologies with a selection of references summarizing research progress and challenges in applying miniature bioreactor systems. It reflects not only on miniaturization concepts but also on 3D printing techniques, as well as on the auxiliary equipment that is part of the screening system. 

## 2. Miniaturization

### 2.1. Scale-Down Concept

**Figure 1:**
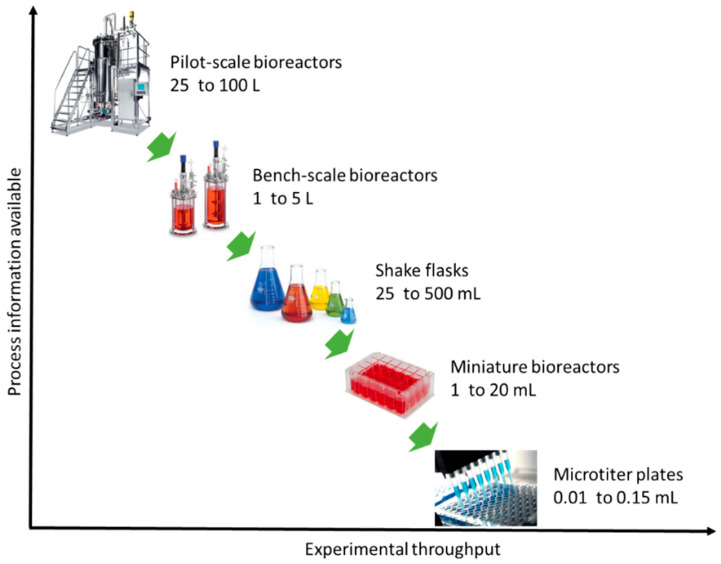
Relation of process information with experimental output for different scale bioreactors.

**Figure 2:**
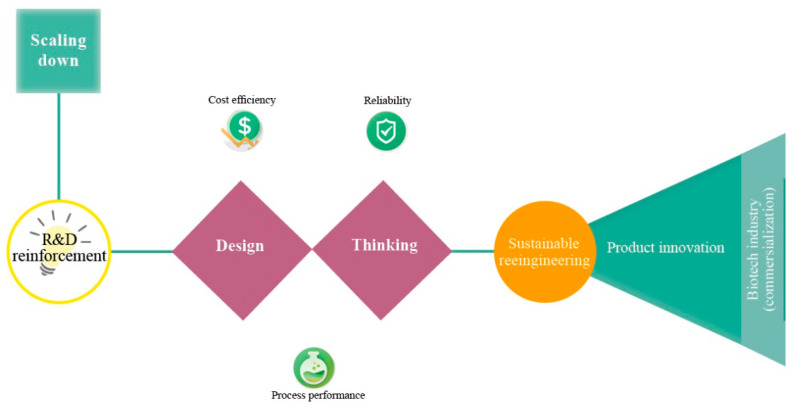
Engineering pathways of the miniaturization concept (top left to right).

According to Latterman and Buchs [14], the design of a bioreactor typically varies in shape, material properties and instrumentation, which have a direct influence on the performance. Miniaturization or else scaling-down of bioreactors is a current trend and promising solution for optimization studies in biotechnology (e.g., fermentation, anaerobic digestion) [15,16]. Miniaturization aims at replacing bench-scale bioreactors and ultimately pilot-scale bioreactors (Figure 1) [17]. Unfortunately, the current small-scale lab systems (e.g., shaken flask systems) lack automated feeding, pH and/or oxygen control, a fact that is unfavorable for developing microbial fermentations [18,19]. Thus, the parallel running miniature system has become a more attractive method as they provide early-stage process understanding during process development [20]. Miniature bioreactors must mimic conditions that microbial communities or pure cultures experience in larger vessels [21,22]. Figure 2 depicts important aspects that have to be considered for the optimal bioreactor design. Reliability, cost-efficiency and process performance are the utmost important aspects of bioreactor design, followed by sustainable reengineering (reactor and process redesign) and product innovation.

Biotech enterprises have to compile current research and frontline developments in bioreactors and make endeavors towards the design and application of sustainable principles though process intensification.

### 2.2. Miniature Bioreactors

In the case of an anaerobic fermentation process, small anaerobic digesters (AD) can serve as a screening tool for biogas production. A fermentation screening system using low-volume reactors would be able to operate and control many parallel fermenters. Besides, parallelization of a miniature AD system enables the monitoring of multiple operational parameters allowing detailed process insight [17]. Currently, no minimum size of a commercial miniaturized bioreactor system has been established. The operating volume of miniature systems preferably remains less than 20 mL. An overview of the state-of-the-art miniature bioreactor systems and their main characteristics is given in Table 1. The control of miniaturized bioreactor systems remains very complex and lacks research. Due to the necessity of an anoxic environment, the downscaling of anaerobic digesters is more challenging compared to other bioreactors, although mixing becomes less demanding. 

**Figure 3:**
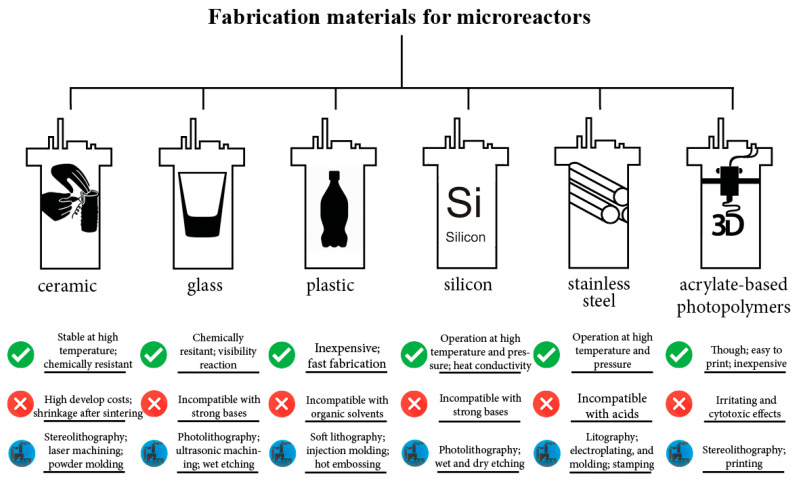
Highlighted features for various miniature reactor fabrication materials [38,39,40,41,42,43,44,45,46,47,48].

The materials employed for the manufacturing of the bioreactors may influence their applicability (Figure 3). The thermal properties of the material, e.g., thermal conductivity, thermal expansion as well as melting point, are important when the reactor vessel is exposed to high temperatures (121 °C). The chemical properties, such as chemical resistance, are also crucial for the operation procedure. Lastly, the physical properties of surfaces affect the reactor vessel performance as they are related to bacterial adherence and biofilm formation [37]. 

A continuous mini-bioreactor system may consist of multiple parts and the reactor vessel is one of those parts. When scaling down the bioreactor, three main aspects must be taken into account. These aspects are maintaining the possibility to sterilize the reactor, maintain perfect mixing and options to measure and control, pH, dissolved oxygen (DO), temperature and level. These three aspects have to be reevaluated when designing a continuous miniature bioreactor. Currently, relatively large and expensive bioreactors are used in the laboratory. If cheaper and smaller bioreactors could replace those bioreactors, while still maintaining the same performance, that would be ideal. A morphological overview is depicted in Table 2 to give an overview of the possible solutions per subfunction. This overview provides a systematic approach to combining solutions and deriving working structures.

### 2.3. Fluid Dynamics

The flow characteristics in a miniaturized bioreactor differ significantly from those in the laboratory scale reactor [49,50]. At the microscale, different forces become dominant over those experienced in everyday life [51]. Because of scaling, it is often counterproductive to simply shrink an existing large device and expect it to function properly at the micro-level [52]. Therefore, new designs must be created to take advantage of forces that work on the microscale. Beebe et al. [49] describe different effects that become dominant in microfluidics, including laminar flow, diffusion, fluidic resistance, surface area to volume ratio, and surface tension. The small dimensions of miniaturized bioreactor systems typically result in laminar fluid flow conditions of the fluid phase [53,54,55,56]. A cylindrical reactor design, which is typical for continuously stirred tank reactors (CSTRs), enhances the predictability of the laminar flow dynamics [57]. The rounding of the edges enables the flow to remain laminar and predictable, rather than becoming a random and complicated flow structure of interacting vortices [51]. Fluid dynamics have successfully integrated different stirring mechanisms and pumps to achieve homogenous conditions [56,58,59,60,61].

### 2.4. Parallelization

Gathering comprehensive process information concerning the development of a bioprocess requires several simultaneous experiments. Therefore, there is a demand for cost-effective, parallel, and multiparametric systems with a high throughput [62]. Parallelization, in bioprocess development, signifies the practice where multiple reactors are employed side by side, thus in parallel [4]. This utilization of multiple reactors in parallel, rather than one reactor, which is used sequentially, increases the experimental throughput. Additionally, the time required for the process development will reduce [2]. By setting the tested parameter differently for each reactor, high experimental throughput is achieved regarding that parameter.

### 2.5. Sensor Capabilities

Online and in real-time monitoring of a bioprocess is inherently complex. However, it ensures the control of the vessel conditions and the optimal use of raw materials. Thus, consistent quality of the final product and a reduction in wastes and process cycle time can be reassured. Besides, the replacement of costly and slow laboratory testing (screening systems) opens up the possibility of bioprocess innovation [12,63]. When the bioreactors are scaled down, the sensors are also required to decrease in size, to assure this detailed monitoring of the bioprocess in the miniature bioreactor. A solution is integrating the sensor into small fabricated devices, which is indicated by [23]. Currently, most of these microfabricated devices with integrated sensing capabilities solely monitor just the basic culture conditions, such as temperature, DO, pH and optical density [62,64]. Besides sensors that are integrated into miniature bioreactors, other available micro-sensors can be used. Examples are MEMS-based chemical concentration sensors [65], gold-plated microscopic electrode needle arrays [66] and miniaturized gas sensors [67]. 

## 3. Facets of 3D Printing

### 3.1. 3D Printing—Additive Manufacturing

**Figure 4:**
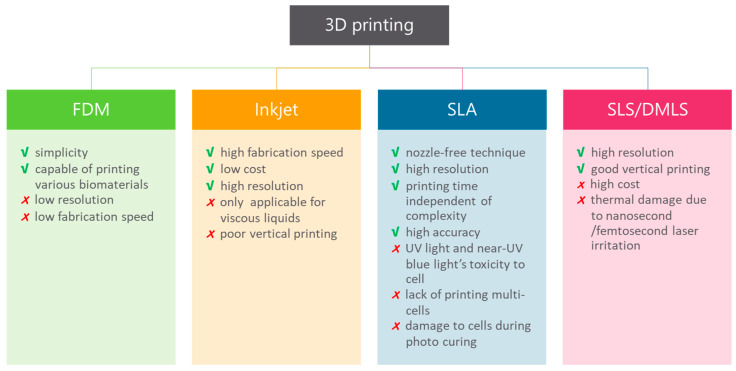
Advantages and limitations of 3D printing techniques. These techniques fabricate an object one layer at a time and include fused deposition modeling (FDM), inkjet bioprinting, stereolithography (SLA), laser sintering (SLS) and direct metal laser sintering (DMLS) [77,78,79,80,81,82,83].

The prospect of fabricating objects with the use of 3D printing has seen increased interest in recent years [12]. Although the range of commercial products is still limited, 3D printing has potential when considering design and fabrication. The potential of 3D printers explains the interest of multiple research fields in 3D printing applicability. To date, 3D printing in life sciences is mainly used for medical applications, but it has attracted the interest of researchers [68,69]. Essentially, 3D printing is an additive manufacturing technique, which means that the object is fabricated layer-by-layer rather than through molding or subtractive techniques like milling or turning. The variety of materials used in 3D printing (e.g., plastic, stainless steel, ceramics, glass, paper, photopolymers and even living cells) ensures opportunities for multiple applications [70,71,72,73,74]. These materials are in the form of powders, filament, liquids and sheets as a starting product. Furthermore, there are multiple techniques used in 3D printing and their advantages and drawbacks are outlined in Figure 4. 

It is found that different types of applications require different kinds of materials and various kinds of 3D printing techniques. Fused deposition modeling (FDM) is a method that uses a polymer as the main material and builds parts with a layer-by-layer-technique from the bottom to the top, by heating and extruding a thermoplastic filament [75]. Benefits are low cost and simplicity of the process. However, the results have weak mechanical properties, layer-by-layer appearances, poor surface quality, low speed and a limited number of thermoplastic materials that can be used [76]. Therefore, this type of 3D printing is not suitable for all applications. Stereolithography (SLA) is another form of 3D printing technology that uses photopolymerization, which is the curing of photo-reactive polymers (resin) by using a visible or ultraviolet laser [75]. A thin layer (25–100 µm) of resin between the bottom of the resin reservoir and a support is cured by illumination with the laser according to a cross-section of the object that needs to be printed. In the next step, the support lifts the object and new resin will flow underneath the first layer that is cured. The next cross-section of the object is illuminated, and the “drawing” process repeats until the object is printed. The unreacted resin is removed after completing the printing. Drawbacks of this method are that it is relatively slow and more expensive than FDM. However, the results are high-quality parts at a fine resolution [76]. 

Besides the pros and cons, 3D printing in general has some significant advantages over other conventional constructional methods. The process of designing and fabricating an object overtakes some traditional manufacturing steps, including procurement of individual parts, creation of parts using molds, machining to carve parts from blocks of material, welding metal parts together and assembly [12]. Another main advantage of 3D printing is the efficiency in which it uses its material. In other words, 3D printing can not only fabricate internally complex objects that are difficult or impossible to produce by traditional manufacturing techniques, but it can also create these objects with fewer wasted materials [12]. On the other hand, 3D printing has some serious limitations, but some of them have been overcome in the last couple of years. These limitations consist of the relatively slow-building speed, limited object size and detail (resolution), high materials cost and, in some cases, limited object strength (depending on which 3D printing technique) [12].

To show the increasing interest in 3D printing, an overview of the articles found on this subject is given on the Web of Science (Web of science.2020). The search terms were 3D printing, 3D printing + reactor and 3D printing + materials, and the search was done on the topic of the publications. An overview is given in Table 2. Most notable is the increase in the number of articles on all different search terms. A vast difference appeared between the number of papers found in 2009 and 2019 about 3D printing. 

**Figure 5:**
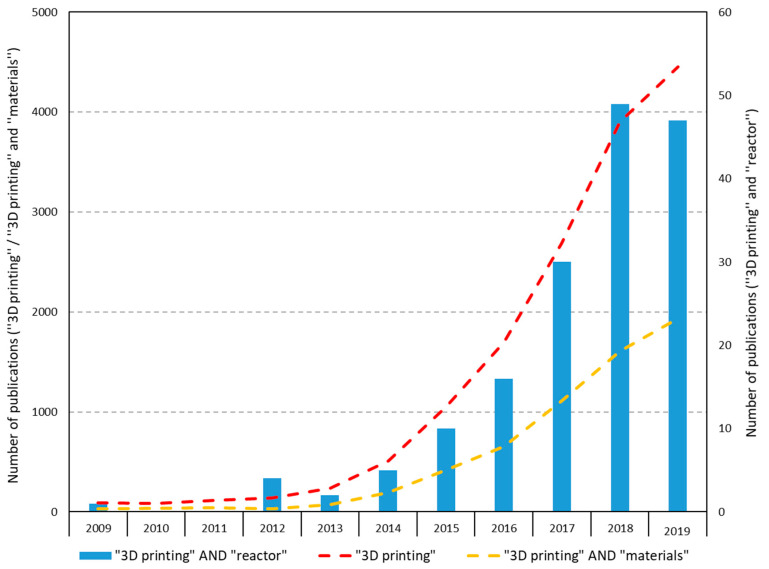
Science citation index publications on 3D printing from the web of science.

However, the combination of “3D printing” and “reactor” is still not that common in the scientific literature. Figure 5 shows that there is an increase, but it is yet to be further explored. This means that a lot of research can and will probably be done in this area and also shows the need of this study.

### 3.2. 3D Printed Bioreactors

The volumes of the 3D printed reactors vary widely: some are about 2.65 μL for the preparation of perovskite nanocrystals [84], while others are 330 mL for chromatography [85]. This shows that 3D printing can be applied to many different applications in many different sizes (Table 3). The conditions also range widely between 37 °C for immobilizing enzymes in hydrogel lattices [86] and an injector temperature of 200 °C for gas chromatography [85]. Two of the found researches are about bioreactors; one of them is used for mechanical stretching and tissue engineering. The volume of this reactor is 1.35 mL, it is printed with FDM and it is fabricated of acrylonitrile butadiene styrene (ABS). There were no malfunctions during testing [87]. The second bioreactor was also manufactured of ABS and it has a volume of 129.9 mL. The application is maintaining cells and engineered tissue in culture medium and custom grips for mounting 3D engineered tissue constructs and soft tissues. The device is sterilized with 70% ethanol but can only withstand a maximum failure load of less than 10 Newton [88]. A mixed flow reactor has been 3D printed with SLA using Clear Resin of Formlabs with a volume of 25 mL [89]. It was used for measuring mineral precipitation rates and can also be modified for use in mineral dissolution experiments.

### 3.3. Biocompatibility

Williams [95] states that biocompatibility refers to “the ability of a material to perform with an appropriate host response in a specific situation”. However, this definition is argued to be so general and so self-evident that it is not of any real help in advancing knowledge of biocompatibility [96]. Therefore, Williams [97] redefined the definition of biocompatibility as “the ability of a biomaterial to facilitate the most appropriate cellular or tissue response, while performing its desired function concerning a medical therapy, while optimizing the clinically desired performance of the therapy, without drawing out any undesired local or systemic effects in the beneficiary or recipient of the therapy”. Black et al. [98] separated the definition of biocompatibility into a host response and a material response. The host response is defined as “the local and systemic response, other than the intended therapeutic response, of living systems to the material”, whereas the material response is defined as “the response of the material to living systems” [98]. These definitions imply that biocompatibility phenomena associated with a biomaterial will vary depending on the application, meaning that biocompatibility is not a property of material but a biomaterial-host system [97]. Based on the generic biocompatibility pathways described by Williams (2014) [99], three main biocompatibility goals are defined as “defensive”, “target” and “interfering”. 

For this particular research, biocompatibility is described by translating the three goals into specific requirements: (1) the material surface area of the bioreactor has to be inert; (2) must be non-biodegradable; and (3) bacteria must not adhere to the material. All three requirements are important for research reproducibility and research integrity and these requirements should be accurately tested and at all times kept in mind when doing microbial experiments.

In the microbial research, upcoming 3D printing techniques show opportunities for biomaterials to fabricate miniature bioreactors. The interactions between microbes and biomaterial influence the functionality of the bioreactors in terms of microbial growth. For the majority, this description can be related to the definition of biocompatibility. Instead of having a local host in the system, the system contains an inoculum or medium in which the microbes interact with the material substrate surface. Now the question remains, is the general definition of biocompatibility limited to the medical point of view and should the definition be overhauled or rephrased, or should another term be used to describe the interactions that happen in microbial research?

### 3.4. Evaluation of Fabrication Techniques

Pahl and Beitz (2007) [100] distinguished two main types of criteria, namely, technical and economic criteria. Both criteria consist of multiple aspects that can be modified to the product or process under consideration. In general, the majority of the conventional bioreactors are fabricated using glass, plastic or metal (Eppendorf, 2018). The most generic and applicable manufacturing techniques concerning these materials are injection molding, casting and milling and turning [101]. The technical criteria cover the performance of the process to the final desired product. The overall quality and lifespan are, therefore, included in the technical criteria aspects. Additionally, it is relevant to how complex and accurate the process is. Thus, how complicated the overall process of producing the product is and how detailed the product can be produced. Lastly, the maximum object size can be included as a technical criteria aspect. Currently, the 3D printers entail a limited object size that can be produced. This should also be considered when evaluating the technical performance.

The economic criteria firstly consider the costs. The costs can be separated into raw material costs, production costs and general overhead costs [102]. Additionally, the time required to produce one product is considered. Lastly, the flexibility of the production process entails is considered in the economic criteria aspects. The information for the fabrication process can be obtained using 3D-printing software (e.g., Formlabs, 3D-simplify or Ultimaker Cura). The software calculates the resources and time required per component. Table 4 shows indicative technical and economic criteria that have to be considered for the selection of a reactor fabrication technique. 

## 4. Conclusions

In this paper, we discussed several important topics on scaling-down and manufacturing of bioreactors. We brought up issues for combining miniaturization and additive manufacturing techniques, culminating in 3D printing, and how this combination can affect the evolution of biotechnology through the fabrication of advanced screening systems. These topics include the future of the 3D printing application in bioprocess technology, potential for bioreactor miniaturization and concurrent development of bioreactor screening systems for optimization studies. These topics are essential for academics, entrepreneurs and policy makers to be aware of and consider as we usher in a new bioprocess renaissance.

## Figures and Tables

**Table 1:** Miniature bioreactor systems and their main characteristics.

Reactor Volume	Application	Material	Mixing	Sensors	Ref.
μBR (150 μL)	Microbial fermentation	PMMA ^1^, PDMS ^3^	Magnetic	pH, DO ^2^	[23]
μBR (150 μL)	Fermentation	PDMS	Peristaltic oxygenating mixer	pH, DO	[24]
μBR (150 μL)	Cell cultivation	Plastic	Unknown	pH, DO, dCO_2_	[25]
Milliliter scale tank BR (10 mL)	Mycelium forming	PEEK *	Magnetic	pH, DO	[26]
Milliliter scale BR (12 mL)	Measuring power consumption/energy dissipation		magnetic	Torque, particle size	[27]
SimcellTM (1 mL)	Cell cultivation		Sparging	pH, DO	[28]
MA (0.1–2.0 mL)	Controlling cellular microenvironment	PDMS	Unknown	Flow velocity	[29,30]
ambrTM (15 mL)	Cell cultivation		Sparging	pH, DO	[31]
ambrTM (15 mL)	Cell cultivation		Sparging	pH, DO	[32]
Mini bioreactor (30 mL)	Mammalian cell culturing		Angled disc impeller	Temperature	[33]
BioREACTOR48 (8–15 mL)	Parallel fermentation		Autoinduction impeller	pH, DO	[34]
RoboLector (800–2400 µL)	Parallel fermentation		Shaking	biomass, pH, DO, fluorescence	[35]
micro-Matrix (10 mL)	Parallel fermentation		Shaking	pH, DO, Temperature	[36]

^1^ Polymethylmethacrylate; ^2^ Dissolved oxygen; ^3^ Polydimethylsiloxan; * Polyetheretherketone.

**Table 2:** Morphological overview within the first column, the subfunctions with multiple suitable solutions and in the remaining columns the different solutions.

Solutions	1	2	3	4	5
Supply thermal energy	Water bath	Water jacket	Hot air oven	Micro heaters	Coil
	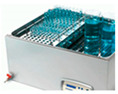	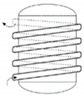	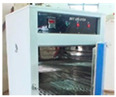	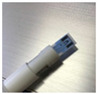	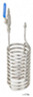
Supply electrical energy	Plug and socket	Battery	Controller		
	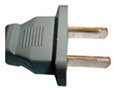	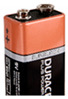	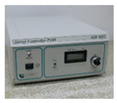		
Supply substrate	Infusion pump	Infusion controller	Effluent and pump	Piezoelectric pressure sensor	
	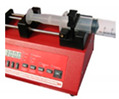	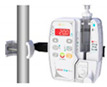	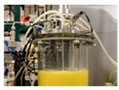	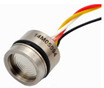	
Open / close reactor	Insertable lid	Lid and thread			
	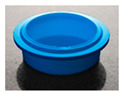	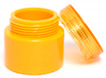			
Ports sealing	Teflon tape	Butyl rubber and needle	Tight thread	Rubber O-ring	1-component design
	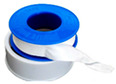	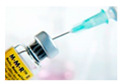	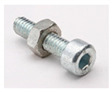	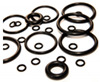	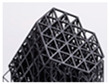
Measure pH	Online mini electrodes	Offline assay kit (pH test strips)	Offline assay kit (fluorometric intracellular pH)		
	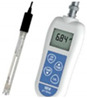	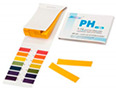	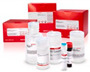		
Measure biogas volume	Gas counter	Water displacement	Syringe	Flow meter	
	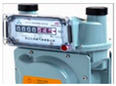	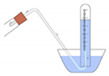	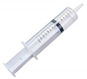	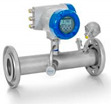	

**Table 3:** 3D printed (bio)reactors and their applications.

Type of Reactor	3D Printing Technique	Printing Material	Volume (in mL)	Conditions	Application	Remarks	Ref.
Enzyme Reactor Paper Spray		Autoclavable polylactic acid plastic	3.5	Heated to 40 °C for 15 min, then 37 °C for 10 min. Then a voltage of 4 kV. Then heated to 68 °C for 5 min.	BuChE detection using a paper strip coated with 4-mercaptobutyrylcholine-functionalized gold nanoparticles	Easy preparation, low-cost, facile modification. High reliability and repeatability	[90]
Mechanical Stretching Bioreactor	FDM	ABS plus-P430 in combination with SR30 soluble support material	1.35	Procedures of a cell biology/tissue engineering laboratory. Laminar flow, mechanical stimulation	Mechanical stretching, tissue engineering	No malfunctions during testing	[87]
Mechanical bioreactor		Acrylonitrile butadiene styrene (ABS)	129.9	Cycle tensile strains are applied. Force and displacement data collection with ramp control program	Low-cost culture chamber for maintaining cells and engineered tissue in culture medium and custom grips for mounting 3D engineered tissue constructs and soft tissues	Can be sterilized with 70% ethanol. Maximum failure loads of less than 10 Newton	[88]
Continuous flow reactor	SLA	Methacrylate photopolymer resin	0.00265	Stirring at 800 rpm	Preparation of perovskite nanocrystals in the full-emission range		[84]
CuO-nanoparticle functionalized flow reactor	FDM	Poly(lactic acid) filaments	0.868	pH 10, reaction temperature = 50 °C, reaction medium = 100 mM phosphate-buffered saline	Online fluorometric monitoring of glucose	The 3D printed flow reactor has several advantages over the conventional flow reactor	[91]
Hydrogel-based enzyme reactor	Pneumatic extrusion-based printing	PEO and Laponite RD	0.507	T = 37 °C, pH 9. Centrifugation with 10,000 rpm, 4 min.	Immobilization of enzymes in hydrogel lattices under mild conditions	Mass transfer limitations occur	[86]
Continuous reactor	FDM	Acrylonitrile butadiene styrene (ABS)	0.15	T = 60 °C, pH 10. Agitation at 400 rpm. Then centrifuged at 5500 rpm for 30 min.	Continuous precipitation of hydroxyapatite nanoparticles for potential tissue engineering applications		[92]
Microfluidic reactor	SLA	Clear methacrylate-based resin	1.008	Plasma samples added, incubated at 56 °C for 15 min.	Carrying out extraction, concentration and isothermal amplification of nucleic acids in a variety of body fluids	Cost-effective scalability. PEG-coating resulted in the best results. Suitable for all types of detection	[93]
Tubular bent reactor	FDM	Polylactic acid (PLA)	330	Injector T = 200 °C. Gas chromatography	Redox-initiated continuous emulsion copolymerization of styrene-butyl acrylate and vinyl acetate-neodecanoic acid vinyl ester	Narrow residence time distribution, small dead volumes and suitable flow characteristics for emulsion copolymerization processes	[85]
Mixed flow reactor	SLA	Clear Resin (Formlabs)	25	Curing treatment	Measure mineral precipitation rates	Can also be modified for use in mineral dissolution experiments	[89]
Miniaturized polypropylene reactor	FDM	Polypropylene	0.25	Magnetic stirring. Infusion rate of 125 μL min^–1^	Online analysis of a Diels-Alder reaction and the subsequent retro Diels-Alder reaction	Resistant to inorganic and organic reagents and solvents	[94]

**Table 4:** Indicative technical and economic criteria for manufacturing technique of reactors [101,103,104,105,106].

Technique	Technical	Economical
3D printing	Quality	Raw material costs
Injection molding	Lifespan	Production costs
Casting	Process complexity	Production time
Milling and turning	Process accuracy	Flexibility
	Quality	General overhead costs
